# The Influence of Electric Field and Confinement on Cell Motility

**DOI:** 10.1371/journal.pone.0059447

**Published:** 2013-03-26

**Authors:** Yu-Ja Huang, Justin Samorajski, Rachel Kreimer, Peter C. Searson

**Affiliations:** 1 Department of Materials Science and Engineering, Johns Hopkins University, Baltimore, Maryland, United States of America; 2 Johns Hopkins Physical Sciences in Oncology Center and the Institute for Nanobiotechnology, Johns Hopkins University, Baltimore, Maryland, United States of America; 3 Department of Chemistry, University of Dallas, Irving, Texas, United States of America; University of Cambridge, United Kingdom

## Abstract

The ability of cells to sense and respond to endogenous electric fields is important in processes such as wound healing, development, and nerve regeneration. In cell culture, many epithelial and endothelial cell types respond to an electric field of magnitude similar to endogenous electric fields by moving preferentially either parallel or antiparallel to the field vector, a process known as galvanotaxis. Here we report on the influence of dc electric field and confinement on the motility of fibroblast cells using a chip-based platform. From analysis of cell paths we show that the influence of electric field on motility is much more complex than simply imposing a directional bias towards the cathode or anode. The cell velocity, directedness, as well as the parallel and perpendicular components of the segments along the cell path are dependent on the magnitude of the electric field. Forces in the directions perpendicular and parallel to the electric field are in competition with one another in a voltage-dependent manner, which ultimately govern the trajectories of the cells in the presence of an electric field. To further investigate the effects of cell reorientation in the presence of a field, cells are confined within microchannels to physically prohibit the alignment seen in 2D environment. Interestingly, we found that confinement results in an increase in cell velocity both in the absence and presence of an electric field compared to migration in 2D.

## Introduction

The asymmetric distribution of ion channels and pumps between the apical and basal surfaces of the endothelial cells surrounding most organs leads to a transendothelial (or transepithelial) potential difference Δφ ( = φ_apical_ – φ_basal_) of +15 to +60 mV, corresponding to a dc electric field of 0.5–5 V cm^−1^ [1]–[3]. This is a relatively small field, about six orders of magnitude lower than the threshold field for electroporation of a cell membrane (approx. 2×10^6^ V cm^−1^) [4]. However, epithelial and endothelial cells are programmed to sense and respond to dc electric fields (dcEFs) in processes such as wound healing, development, and nerve regeneration [1]–[3], [5]–[8]. Electric fields are also thought to play a role in angiogenesis [3] and metastasis [1].

In cell culture, electric fields influence cell division, polarity, shape, and motility. Many cell types respond to dcEFs, preferentially migrating either to the anode or cathode, a process known as galvanotaxis [2]. *In vitro* studies of galvanotaxis are usually performed in 2D by analyzing the path traced by individual cells in the presence or absence of an electric field. Most cell types respond to dcEFs of the magnitude of endogenous electric fields, however, the origin of this directionality and the mechanism of galvanotaxis remain unknown.

For many processes of physiological interest cell motion is physically confined. For example, during migration through the extracellular matrix, cells migrate along channels between bundled collagen fibers [9]. Similarly, during wound healing, cells must squeeze between other cells. Various cell migration chambers have been developed for the study of galvanotaxis in 2D [10]–[15], including microfluidic-based platforms [12], [14], [15]. Our objective is to develop a versatile microfluidic-based platform to study galvanotaxis in 2D and confined geometry.

Here we report on quantitative characterization of the physical and morphological aspects of the motility of NIH 3T3 fibroblasts under an electric field and physical confinement. Although galvanotaxis is usually associated with directional bias towards the cathode or anode we show here that the influence of dcEFs on motility is much more complex. In 2D (no confinement) and in an electric field, cells orient perpendicular to the field vector and migrate preferentially towards the cathode. Unexpectedly we show that the electric field exerts forces on the cells both parallel and perpendicular to the field. These forces are in competition with each other and ultimately govern the trajectories of the cells in the presence of an electric field. At low field, the cells migrate preferentially towards the cathode, however, the perpendicular component of the individual segment vectors is larger than the parallel component. In a larger field, there is a significant increase in average velocity and the parallel component of the individual segment vectors is larger than the perpendicular component. These results suggest that there could be at least two independent signaling pathways that influence cell motility in an electric field. To further probe the effect of perpendicular alignment on directed migration induced by the electric field, 3T3 cells were seeded inside 20 µm channels to physically prevent cell orientation during galvanotaxis. We found that physical confinement results in an increase in cell velocity both in the absence and presence of an electric field compared to migration in 2D. This result could be relevant in understanding galvanotaxis *in vivo*.

## Materials and Methods

### Microfluidic Platform

**Figure 1:**
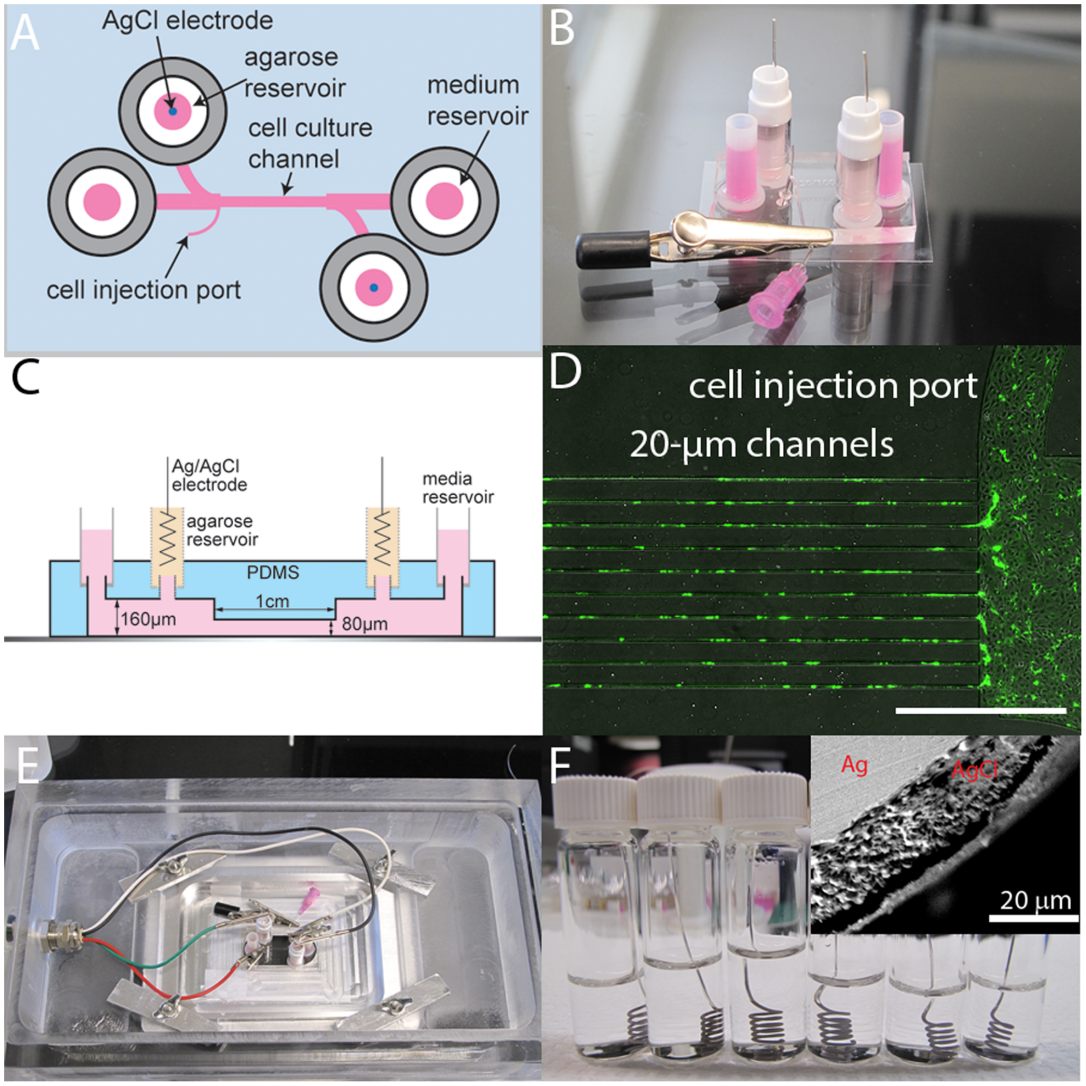
Microfluidic platform. (A) Schematic illustration of the galvanotaxis platform for the study of galvanotaxis in 2D and under physical confinement. (B) Photograph of the platform on a 35×50 mm glass slide. (C) Cross-sectional illustration of the galvanotaxis platform. (D) Fluorescence image of a device with ten 20 µm channels. Prior to seeding cells in the device, the internal surfaces of the channels are coated with fibronectin to mediate cell-substrate interaction. FITC-conjugated fibronectin (green). (E) Photograph of the device in a live-cell chamber. (F) Photograph of AgCl electrodes fabricated by chloridization of silver wires. Inset: cross section SEM image showing the 20 µm AgCl layer on the silver wires.

There are two key features of our platform (Fig. 1). (1) Silver chloride electrodes are integrated into the platform without the need for external salt bridges and solution reservoirs. By integrating the electrodes into the microfluidic platform, our device is sufficiently small to fit in a live cell chamber (Figs. 1D and 1E) so that experiments can be performed under controlled relative humidity, CO_2_ partial pressure, and temperature. As described below, by using silver chloride electrodes, the only species involved in the electrochemical reactions at the electrode surface is the consumption or generation of chloride ions. In this way, we can avoid the parasitic reactions associated with noble metal electrodes such as gold or platinum. (2) By controlling the width w of the channels relative to the cell diameter d_cell_ we can study cell migration under an electric field in 2D (w>>d_cell_) or in confined dimensions (w≤d_cell_). Such quasi-1D migration is analogous to migration along fibers in 3D gels, such as extracellular matrix [16], [17]. Confinement experiments were performed in 20 µm, 50 µm, and 100 µm wide channels. Here we compare cell migration in 2D (w×h, 1000 µm×80 µm) and in 20 µm channels (w×h, 20 µm×80 µm); experiments in 50 µm and 100 µm channels showed intermediate behavior and hence did not provide any additional insight. The details of platform fabrication are provided in Supporting Information.

### Fabrication of Ag/AgCl Electrodes

The electric field was applied between two Ag/AgCl electrodes located at each end of the microfluidic channel (Fig. 1). Ag/AgCl electrodes have the advantage that the equilibrium at both electrodes is established by the reaction AgCl+e^−^ ↔ Ag+Cl^−^ and that the current is carried by chloride ion transport. The electrodes were fabricated by electrochemically forming AgCl on two inch lengths of 0.025 inch diameter silver wire (A–M systems, Sequim, WA) using standard procedures [18]. Briefly, the silver wire and a platinum foil cathode were immersed in 1M HCl solution and chloridized for 30 minutes at a current of 5–10 mA cm^−2^. The wires were then removed from the HCl solution, rinsed and stored in distilled water (Fig. 1F). The electrodes were coiled to ensure that about two inches was embedded in the agarose gel (see *Supporting Material* for details). From scanning electron microscope images (Fig. 1F), we determine an average AgCl thickness of 20 µm. A current of about 75 µA is required to maintain a field of about 5.5 V cm^−1^ within a 1 cm long and 1000 µm wide channel (cross sectional area of 0.08 mm^2^), and from Faraday’s law, we determine that a 5.5 V cm^−1^ field can be maintained for more than six hours. Experiments were performed in the absence of a field, and with an electric field of 2.2 V cm^−1^ or 5.5 V cm^−1^. The magnitude of the field was confirmed using a four point probe method with two platinum wires inserted at each end of the channel.

### Maintenance of Cell Lines

3T3 mouse fibroblast cells (ATCC) were cultured in DMEM media with 10 vol.% calf bovine serum (CBS) and 1 vol.% penicillin-streptomycin (PenStrep). Prior to experiments, cells were washed with phosphate buffer (PBS) and lifted from the surface using trypsin (0.25% trypsin-EDTA in PBS, Sigma). Suspended cells were centrifuged and re-suspended in 500–1000 µL of media before introducing into the platform through the cell injection port using a syringe. The cells were introduced at a density of 300,000 cells mL^−1^.

### Imaging

Time-lapse phase-contrast images were collected using an inverted microscope with a 10X objective (TiE2000, Nikon, Melville, NY). A live-cell chamber was used to control temperature (37°C), CO_2_ (5 vol.%), and relative humidity (95%) (Fig. 1E). The device was located in a custom holder placed on an automatic stage in the live-cell chamber. The holder was customized with electrical feed-throughs to connect the electrodes to an external power supply (Fig. 1E). Time-lapse images were taken at 5 min intervals for three hours using NIS-element software (Advanced Research Edition 3.22.11, Nikon, Melville, NY).

### Image Analysis

**Figure 2:**
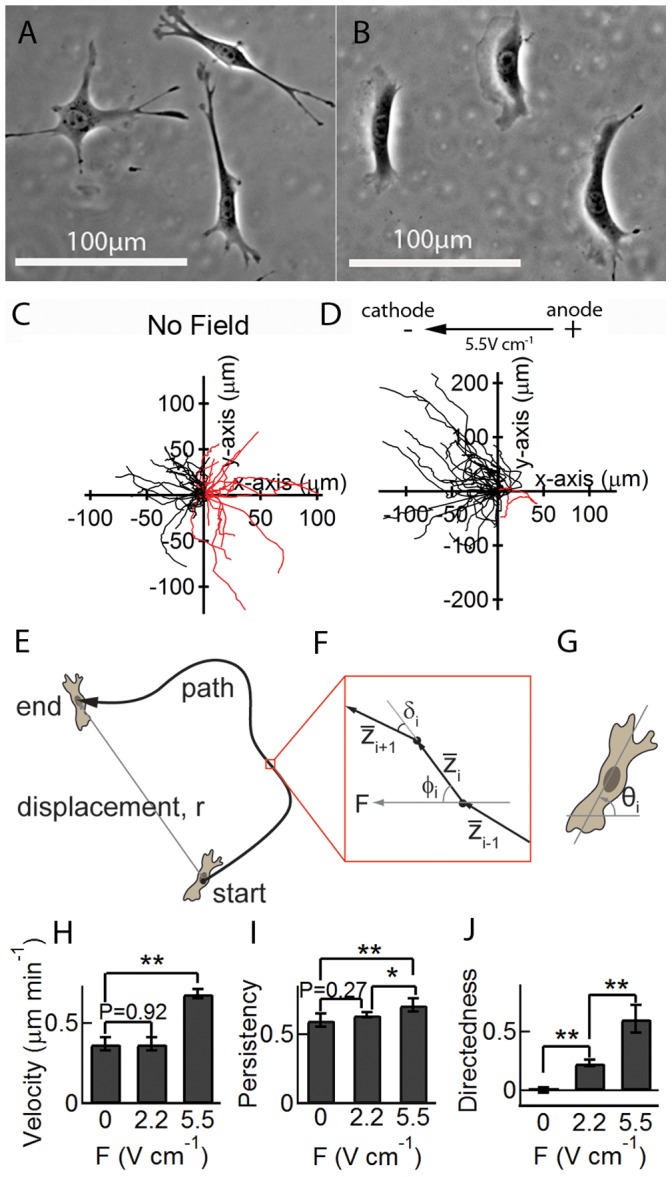
Influence of electric field on the morphology of 3T3 cells and cell path. (A) Phase contrast image of 3T3 cells in 2D with characteristic spindle shape and multiple filipodia. (B) Phase contrast image of 3T3 cells in a 5.5 V cm^−1^ electric field showing perpendicular orientation with respect to the field, significant reduction in filipodia, and broad cathode-facing lamellipodia. (C) Trajectories of 3T3 cells in 2D (no field). Each cell path was analyzed and overlaid at the origin. With no bias, cells have equal probability to travel toward the left (black; 53%) and right (red; 47%) (N = 43). (D) In a 5.5 V cm^−1^ field, the paths are strongly biased toward the cathode (92%, N = 38). (E) Schematic illustration of a cell path. (F) Cell paths are comprised of individual segment vectors (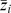) characterized by the angle between the segment vector and the field vector (φ), and the segment turn angle (δ). (G) The cell orientation (θ) is determined by the angle between the long axis and the field axis. (H) The average cell velocity does not change in a 2.2 V cm^−1^ field (P = 0.92) but increases significantly in a 5.5 V cm^−1^ field. (I) The average persistency of the cells also does not change in a 2.2 V cm^−1^ electric field, but increases significantly in a 5.5 V cm^−1^ field. (J) The average directedness of the cells increases with the magnitude of the electric field. *P<0.05; **P<0.01; Student’s t-test. Data were obtained from three independent experiments, with 30–50 cells in each experiment. Error bars indicate standard error.

Time-lapse images were stacked using ImageJ (Version 1.45, NIH, Bethesda, MD) and loaded into Metamorph (Version 7.7, Molecular Devices, Sunnyvale, CA) for analysis of cell paths. Each cell path is made up of individual segments determined by the time between images (Δt = 5 min). Cell paths were only analyzed if the cells did not divide, were not in contact with other cells, and did not leave the field of view. The cell position in each frame was determined from the position of the nucleus. The cell path data was then analyzed using Matlab (MathWorks, Natick, MA). For each cell we determine the persistency, the cell velocity in each segment, the angular orientation of a segment with respect to the field vector, the average directedness, the segment turn angle, and the orientation of the cell with respect to the field vector (Fig. 2E–G).

1. The velocity v(t) is determined from the displacement Δz_i_ and elapsed time between successive images (each segment along the cell path).
2. The persistency (or directional persistence) p is a measure of the linearity of a path and is determined from r/∑Δz_i_ where r is the overall displacement (distance between the start and end points) and ∑Δz_i_ is the length of the path (i.e. the sum of the individual segments along the path). The persistency varies between 0 and 1: p = 0 corresponds to no displacement whereas p = 1 corresponds to a linear path (maximum bias). This term is also known as the directional persistence and in studies of chemotaxis is known as the chemotactic index (CI) [19]. Note that the terminology can be misleading since the persistency does not indicate whether a path is aligned with an external bias, such as electric field or chemoattractant.
3. The directedness d_i_ is a measure of the directional bias of each segment and is given by d_i_ = cos(φ_i_), where φ_i_ is the angular orientation of a segment vector (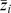) with respect to the field vector and 0°≤φ≤180°. d_av_ = 0 corresponds to random motion whereas values of ±1 correspond to linear motion parallel or antiparallel to the field vector. d_av = _1 (φ = 0°) corresponds to the case where cells move in the direction parallel to the field vector, whereas for d_av_ = ^−^1 (φ = 180°) cell motion is antiparallel to the field. For all experiments in the presence of an electric field, d_av_ represents the average directedness over one hour during steady state motion after the initial transient response.
4. The segment turn angle δ_i_ is defined as the angle between two successive segment vectors, 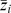 and 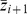 (Fig. 2F), and ranges between 0° and 180°: δ = 0° corresponds to no change in direction whereas δ = 180° corresponds to a complete reversal in direction. By analyzing the distribution of segment turn angles, we can determine the correlation between each step under the influence of a dcEF and under confinement.
5. The cell orientation θ(t) is determined from the orientation of the long axis of the cell with respect to the field axis as a function of time. θ(t) has values from 0°–90° and the average value is expected to be 45° for a random distribution.
6. Analysis of the mean square displacement (MSD) is often used to extract parameters such as the cell velocity and directional persistence. For comparison, details of data processing, analysis and results are provided in Supporting Material and Figure S1.

## Results and Discussion

### Galvanotaxis Alters Cell Morphology and Directs Cell Migration

Many cell types are known to respond to dcEFs with morphological changes and increased motility [20]–[22]. The exposure to dcEFs strongly dictates cell orientation and the direction of migration. In the absence of a dcEF, isolated 3T3 cells display typical fibroblast morphology with an elongated cell body and multiple protrusions (Fig. 2A and Video S1) [23]. However, on application of an electric field, cells orient perpendicular to the field vector and are characterized by the formation of a broad lamellipodium extending the length of the cell and facing the cathode (Fig. 2B and Video S2).

In the absence of an electric field, 53% of the cell paths (N = 43) were pointed in the direction of the field vector (towards the cathode) illustrating that there is no significant directional bias (Fig. 2C). However, in the presence of an electric field, the cell paths are strongly biased in the direction of the cathode (Fig. 2D) consistent with previous reports [22], [24]. In a 5.5 V cm^−1^ field, 92% of the cell paths (N = 38) were directed towards the cathode. The average velocity of 3T3 cells in the absence of a field was 0.38±0.02 µm min^−1^ (SE) (Fig. 2H). In a 2.2 V cm^−1^ field, there was no significant change in cell velocity (0.40±0.03 µm min^−1^ (SE)), but cells preferentially moved toward the cathode and aligned in the direction perpendicular to the field. However, in a 5.5 V cm^−1^ field, the average velocity increased to 0.68±0.03 µm min^−1^ (SE).

The persistency of the cells in the absence of a field was 0.60±0.05 and remained approximately the same in a 2.2 V cm^−1^ field (0.64±0.02 (SE), P = 0.27) (Fig. 2I). Further increasing the field to 5.5 V cm^−1^ significantly increased the persistency to 0.71±0.05 (P<0.01). The average directedness in the absence of a field was −0.02±0.06 (SE). The directedness increased to 0.23±0.03 (SE) in a 2.2 V cm^−1^ field and 0.56±0.12 (SE) in a 5.5 V cm^−1^ field **(** Fig. 2J).

Despite the change in morphology and the increase in directedness of the cell path (Fig. 2J), the average cell velocity under a 2.2 V cm^−1^ field (0.37 µm min^−1^) is the same as experiments with no field (Fig. 2H). A larger field of 5.5 V cm^−1^ results in an increase in the average cell velocity (Fig. 2H) and a further increase in the directedness (Fig. 2J). This result reveals a complex interplay between morphology, cell path, and cell speed, and suggests that cell velocity should not be used as the only parameter to probe the influence of electric field on cell motility.

The average cell orientation was strongly influenced by the field. In the absence of a field, the average orientation was 44.8°±3.45° (SE), whereas at 2.2 V cm^−1^ and 5.5 V cm^−1^, the average orientations were 71.5°±3.1° and 80.3°±0.94°, respectively.

**Figure 3:**
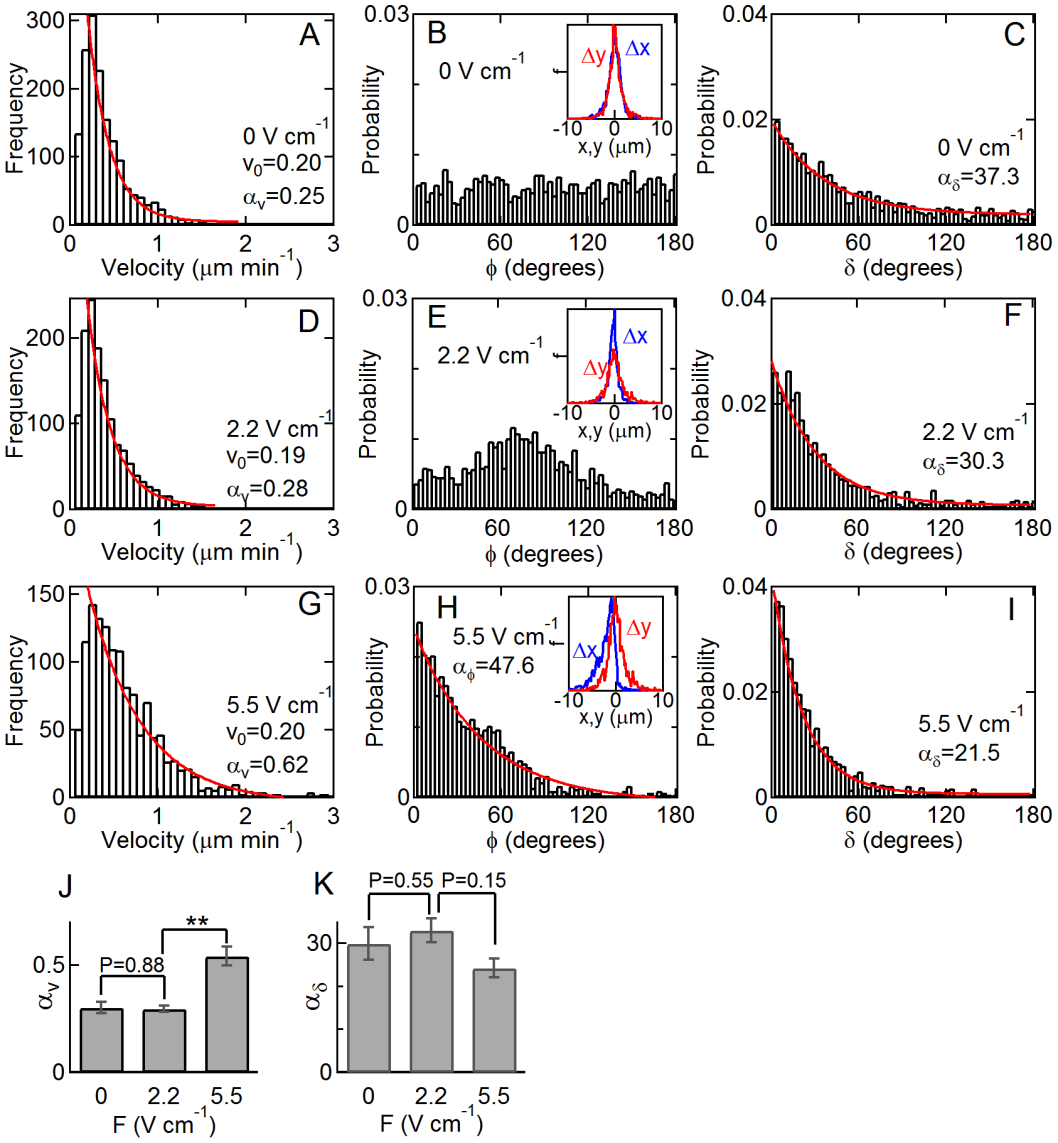
Distributions of average cell velocity, segment orientation (φ), and segment turn angle (δ) in 2D with no field (A, B, C), in a 2.2 **V cm** ^−**1**^ **field (D, E, F) and in a 5.5** **V cm** ^−**1**^ **field (G, H, I).** The distribution of velocity is exponential both in the absence of an electric field (R^2^ = 0.94) (A), in a 2.2 V cm^−1^ (R^2^ = 0.99) (D), and in a 5.5 V cm^−1^ field (R^2^ = 0.94) (G). In the absence of an electric field, φ is uniformly distributed (B) and the distribution of the horizontal displacement (Δx) and vertical displacement (Δy) of individual segments overlap (B, inset). In a 2.2 V cm^−1^ field the perpendicular component of cell motion is larger than the horizontal component as shown by a peak around 70° (E), and thus, Δy has a greater effect on cell motion as indicated by the broader distribution of Δy around zero (E, inset). The distribution becomes exponential in a 5.5 V cm^−1^ field as the parallel component of the bias becomes dominant (H). The distribution of Δx also shows bias toward the negative-x (cathode) direction (H, inset). The distribution of δ is exponential under no field (C) and field (F and I). The solid lines are least squares fits to an equation of the form f/f_0_ = exp(−x/α). (J) The average cell speed coefficient α_v_ increases in a 5.5 V cm^−1^ field. (K) The average segment turn angle coefficient α_δ_ is smaller in the presence of a 5.5 V cm^−1^ electric field but not significant. * P<0.05; ** P<0.01; Student’s t-test. Error bars indicate standard error. Statistics were obtained from at least two independent experiments with 30–50 cells in each experiment. Error bars indicate standard error.

We next analyzed the velocity distributions for the individual segments in a cell path. The cell velocities follow an exponential distribution according to f/f_0_ = exp(−(v−v_0_)/α_v_)), where v_0_ is the most probable velocity (defined by f_0_) and α_v_ is the characteristic velocity. Fits were determined for all velocities greater than or equal to the velocity at the peak of the distribution, v_0_ (Figs. 3A, D and G). In the absence of an electric field, α_v_ = 0.25 µm min^−1^ (R^2^ = 0.94) (Fig. 3A). Although the persistent random walk model for motility commonly used to describe individual cell motility predicts a Gaussian distribution [19], [25], [26], exponential velocity distributions have been reported for various cell types [27], [28], and it has been suggested that this behavior may be due to the limited production rate of ATP [27].

The distribution remains exponential with a similar exponent in a 2.2 V cm^−1^ field (R^2^ = 0.99) (Fig. 3D), however, in a 5.5 V cm^−1^ field, α_v_ increased to 0.62 µm min^−1^ (R^2^ = 0.92) (Fig. 3G). This increase is consistent with the observation that the values of v_0_ are around 0.20 µm min^−1^ in each case and that the average velocity remains the same between 0 and a 2.2 V cm^−1^ field but increases from 0.37 to 0.68 µm min^−1^ between 0 and a 5.5 V cm^−1^ field. The coefficient (α_v_) is proportional to the average cell speed (Fig. S2).

To characterize the orientation of individual segments, we analyzed the angular orientation of the segment vectors and the distributions of the parallel (Δx) and perpendicular (Δy) components of the displacements in each segment. In the absence of an electric field, the angular distribution of segment vectors φ_i_ is relatively uniform with an average value of 42° (Fig. 3B). Thus, the corresponding scatter plot of the x- and y-components of each segment is symmetrical about the origin (Fig. S3A). The distributions of the parallel and perpendicular components of each segment overlap and are characterized by a gaussian distribution centered around zero (Fig. 3B inset). In a 2.2 V cm^−1^ field, a peak around 70° indicates that cells move more in the direction perpendicular to the electric field (Fig. 3E) than parallel to the field. The tendency is further shown in the corresponding scatter plot (Fig. S3B). In the presence of a 2.2 V cm^−1^ field, the distribution of Δy becomes broader and the frequency of Δx is significantly higher than Δy in the range of ±2 µm (Fig. 3E inset), indicating that the average perpendicular component of the displacement is greater than the parallel component. In a 5.5 V cm^−1^ field, the distribution of φ_i_ becomes strongly biased to small angles with respect to the field vector and can be described by an exponential function according to p/p_0_ = exp(−φ/α_φ_), where p_0_ is the probability at φ = 0° and α_φ_ is the characteristic segment angle. From a fit we obtain a coefficient α_φ_ = 47.6° in a 5.5 V cm^−1^ field (R^2^ = 0.89) (Fig. 3H), indicating that most segment vectors are within 48° of the field vector. A 5.5 V cm^−1^ electric field also significantly biased the distribution of Δx towards the cathode making it no longer symmetric around zero (Fig. 3H inset).

The distribution of segment turn angles δ_i_ can be described by an exponential function according to p/p_0_ = exp(-δ/α_δ_), where p_0_ is the probability at δ = 0 and α_δ_ is the characteristic turn angle. In the absence of a field (Fig. 3C), δ is weakly exponential with α_δ = _37.3° (R^2^ = 0.79). The exponent decreases progressively with increasing field with α_δ_ = 30.3° (R^2^ = 0.77) in a 2.2 V cm^−1^ field and α_δ_ = 21.5° (R^2^ = 0.79) in a 5.5 V cm^−1^ field (Figs. 3F and 3I). The segment turn angle and cell velocity distributions remain exponential in the presence of an electric field indicating that the mechanism of motility is the same despite the dramatic change in cell morphology (Figs. 2A–B). Furthermore, there is no obvious influence of the dominant perpendicular contribution to cell motion seen at 2.2 V cm^−1^ (Fig. 3E). It has been suggested that the exponential distribution of segment turn angles reported for the migration of eukaryotic cells is due to the random nucleation of an actin-protein complex anchored to the leading edge of a protrusion [29].

As shown above, the angular orientation (φ) of individual segments is random in the absence of a field (Figs. 3B and S3A) but becomes biased in the presence of a field. In a 2.2 V cm^−1^ field, the distribution of φ becomes biased with a broad peak between 60° and 90°, indicating that the perpendicular component of the bias is larger than the parallel component (Figs. 3E and S3B). Although the cell paths are biased towards the cathode, as seen by the increase in average directedness from 0 to 0.23 (Fig. 2J), the cells are biased to move preferentially perpendicular to the field vector at small fields. Increasing the electric field to 5.5 V cm^−1^ further biased the trajectories toward the cathode, however, the parallel component of cell motion becomes dominant and the distribution of φ becomes exponential (Fig. 3H). The coefficient α_φ_ = 47.6° implies that most segment vectors are within about 45° of the field vector. This can be seen more clearly in the scatter plot (Fig. S3C). These results suggest a complex response to the electric field. There is a global bias towards the cathode, as well as a mechanism that results in motion perpendicular to the field vector at low fields. At high field, the perpendicular component is dominated by a horizontal component parallel to the field vector, resulting in an exponential distribution of segment angles mostly within a characteristic cone of about ±45° around the field vector.

In summary, in response to an electric field, 3T3 cells in 2D are biased towards the cathode. In a small electric field (2.2 V cm^−1^), the average cell velocity and persistency are the same as with no field, however the cells reorient perpendicular to the electric field and are biased towards the cathode. Furthermore, the vertical component of each segment (perpendicular to the field vector) is larger than the horizontal component (parallel to the field). In a larger electric field (5.5 V cm^−1^), the average velocity and directedness increase significantly. In addition, the horizontal component of the segments along a path become larger than the vertical component, and the segment vectors are generally within a cone of about ±45° from the field vector. The distributions of cell velocities and segment turn angles are exponential in all cases. These results suggest that the mechanism of motility is due to a random process within the cell. However, the electric field modulates two processes, one that regulates cell motion perpendicular to the field and one that regulates cell motion parallel to the field. Globally, cell motion is not completely random in that a cell has limited ability to make large directional changes due to the orientation of protrusions associated with the spindle like morphology in the absence of a field and the broad cathode-facing lamellipodia in the presence of an electric field.

### Galvanotaxis is Enhanced Under Confinement

**Figure 4:**
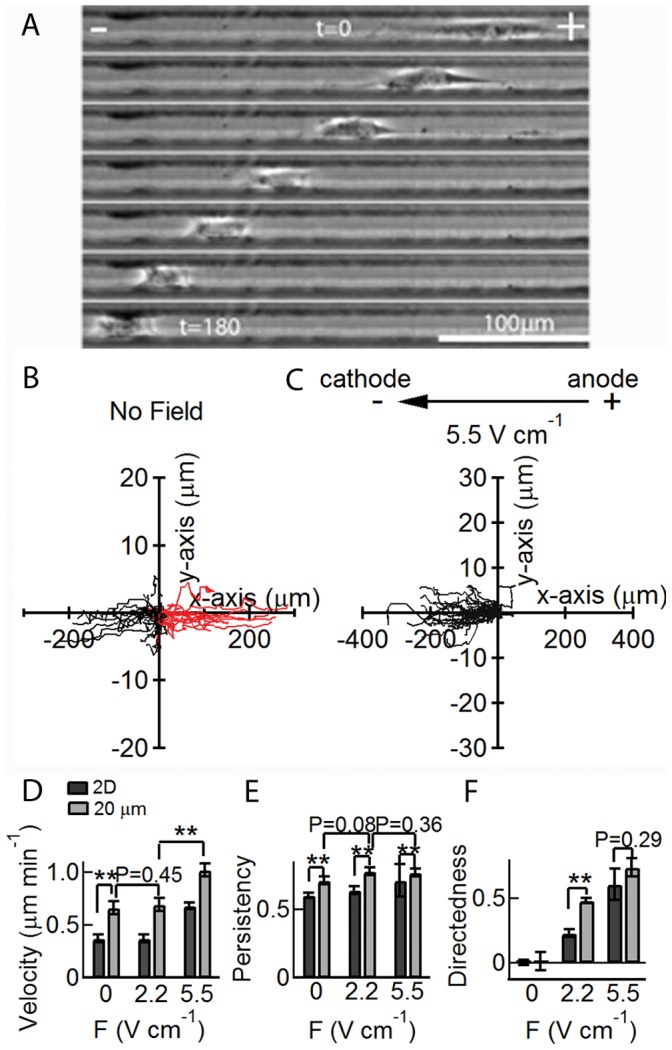
Confinement in 20 µm channels influences galvanotaxis. (A) A series of phase contrast images taken at 30 min intervals showing motion of a cell in a 5.5 V cm^−1^ field. Cell paths with no field (B) and in a 5.5 V cm^−1^ field (C). Note that the axes are not isotropic. In the absence of a field, 53% of the cells moved towards the cathode, whereas in a 5.5 V cm^−1^ field, 100% of the cells moved towards the cathode. (D) In the absence of a field, the average velocity increased under confinement (v = 0.68 µm min^−1^±0.03 S.E.) compared to no confinement (v = 0.37 µm min^−1^±0.04 S.E.). In a 2.2 V cm^−1^ field, there was no change in average cell velocity (P = 0.45). A 5.5 V cm^−1^ field resulted in an increase in average velocity (P<0.01). Black bar: 2D, Gray bar: 20 µm channel (E) Spatial confinement significantly increases the average persistency of the cell paths comparing to no confinement. The influence of electric field on cell persistency is not significant. (F) In the absence of a field, the directedness remained close to zero under confinement. The directedness under confinement increased with increasing field. ** P<0.01. Student’s t-test.

To further study the effects of perpendicular alignment in 2D galvanotaxis on motility, cells are confined within microchannels to prohibit the orientation in the perpendicular direction. In the presence of an electric field and without confinement, cells oriented perpendicular to the field vector with a length of around 100 µm (Fig. 2B), therefore 20 µm wide channels aligned parallel to the field vector prevent the reorientation seen on an unconfined surface. In addition, spatial confinement allows us to impose quasi-1D motion on the cell paths. When confined to 20 µm channels, in the absence of an electric field, 53% of the cells (N = 40) moved in the direction of the field vector although the cell paths were strongly oriented due to the spatial confinement (Fig. 4B and Video S3). In the absence of a field, the average velocity in the channels was 0.68±0.03 µm min^−1^ (SE), significantly faster than cells with no confinement (P<0.01) (Fig. 4D).

Under confinement, cells were forced to align in the direction parallel to the channels. In most cases, cells migrated with the membrane in contact with the channel bottom and the channel walls. In the presence of a field, instead of a broad and persistent cathode-facing lamellipodia, as seen under no confinement (Fig. 2B), cells tended to contract their trailing edge and migrated with a smaller projected area and with the cell body becoming less flat (Fig. 4A and Video S4). In the presence of a 5.5 V cm^−1^ field, 100% of the cells (N = 35) moved in the direction of the field vector. However, as we discuss later, this does not mean that all segments along a path were in the direction of the field vector.

In the presence of a field, cells migrated rapidly in the direction of the cathode (Fig. 4C). For all experiments in 20 µm channels, the average velocity was significantly larger than the corresponding case with no confinement (P<0.01) (Fig. 4D). In a 2.2 V cm^−1^ field, the average velocity was 0.70±0.02 µm min^−1^ (SE), similar to the velocity in the absence of a field (P = 0.45). For a 5.5 V cm^−1^ field, the average velocity was 1.02±0.07 µm min^−1^ (SE), significantly larger than for a 2.2 V cm^−1^ field (P<0.01). The increase in cell velocity in the channels can be due to either the change in cell morphology dictated by the spatial confinement or by the influence of channel walls on motility. An increase in cell speed has also been reported for 3T3 cells on quasi^−^1D lines modified with fibronectin formed by photopatterning and was thought to be due to the suppression of cell spreading and lateral lamellae [30].

The persistency in 20 µm channels was significantly higher than the corresponding case with no confinement (P<0.01). However, the persistency under confinement did not change significantly with electric field. In the absence of a field, the persistency in the channels was 0.71±0.03, whereas in a 2.2 V cm^−1^ field the persistency was 0.78±0.03 (P = 0.08). Further increasing the field to 5.5 V cm^−1^ had no obvious effect on persistency (P = 0.36) (Fig. 4E).

In the absence of an electric field, the average directedness in the channels was 0.01±0.1 (SE), very similar to cell motion without confinement (Fig. 4F). The average directedness increased to 0.48±0.06 (SE) under a 2.2 V cm^−1^ field, significantly higher than experiments without confinement (Fig. 4F). Under a 5.5 V cm^−1^ field, the directedness increased to 0.71±0.04 (SE), very similar to experiments without confinement under the same field (P = 0.29), but significantly higher than for a 2.2 V cm^−1^ field (P<0.05). These results indicate that the influence of confinement is inversely related to the magnitude of the field. In a low field, confinement induces significant directional bias, whereas in a high field the directional bias is dominated by the field.

The velocity distributions remain exponential for motility in 20 µm channels both in the absence and presence of an electric field, indicating that the mechanism governing cell speed is the same in all cases, despite the differences in cell morphology. It has been suggested that cell migration under confinement is less dependent on integrin-mediated adhesion but depends largely on microtubule dynamics [31], [32] and actin polymerization at the cell membrane [33]. Therefore, although physical confinement can induce cytoskeletal alterations, the rate-limiting step that governs cell speed in unconfined and confined geometry may be the limited production rate of ATP, which gives rise to the exponential distribution of cell speed [27]. The distributions were fit using the same procedure as described previously. The coefficient v_0_ was independent of the field, with values of 0.13 µm min^−1^ (no field), 0.13 µm min^−1^ (2.2 V cm^−1^) and 0.14 µm min^−1^ (5.5 V cm^−1^) (Figs. S4A, D, G). The exponents increased with increasing field, with α_v_ = 0.5 µm min^−1^ (R^2^ = 0.88; no field), α_v_ = 0.66 µm min^−1^ (R^2^ = 0.89; 2.2 V cm^−1^), and α_v_ = 1.2 µm min^−1^ (R^2^ = 0.92; 5.5 V cm^−1^) (Figs. S4A, D, G), however, the coefficients are comparable to the values obtained with no confinement (Fig. S4J).

The influence of confinement on cell trajectory was further characterized by examining the angular orientation of the segment vectors and the segment turn angle (Fig. S4). In the absence of a field, the segment orientation angle φ with respect to the field vector was bipolar and symmetrical (Fig. S4B) since cells have equal probability to travel to the left or the right in the channels. The distribution around zero is exponential with α_φ_ = 5.9°. This is in contrast to cell motion on a 2D surface where the distribution of angles is flat (Fig. 3B). In the presence of a 2.2 V cm^−1^ field, the distribution becomes asymmetric due to the bias on the cell paths as shown by the higher probability of φ near zero (Fig. S4E). The distribution around zero remains exponential with α_φ_ = 5.1°. In a 5.5 V cm^−1^ field, the probability of φ near zero further increases and can be characterized by an exponential distribution with α_φ_ = 5.2° (Fig. S4H). However, even though 100% of the cells moved toward the cathode in the presence of a 5.5 V cm^−1^ field, a small subset of segments along a path were pointed in the direction against the field vector as shown by the small peak near φ = 180°.

The segment turn angle, in the absence of a field, is bipolar but asymmetric with low probability around 180° (Fig. S4C). The distribution around zero is exponential with α_δ_ = 7.7°. In the presence of a 2.2 V cm^−1^ field, the distribution becomes more polarized (Fig. S4F) but the distribution around zero remains exponential with α_δ_ = 6.2°. Further increasing the field to 5.5 V cm^−1^ has no significant effect on the distribution of α_δ_ (Fig. S4I) around zero, which remains exponential with α_δ = _7.0°. The segment turn angle coefficients obtained in 20 µm channels are significantly smaller than the values obtained on 2D surfaces (Fig. S4K).

### Transient Response in Directedness and Orientation

**Figure 5:**
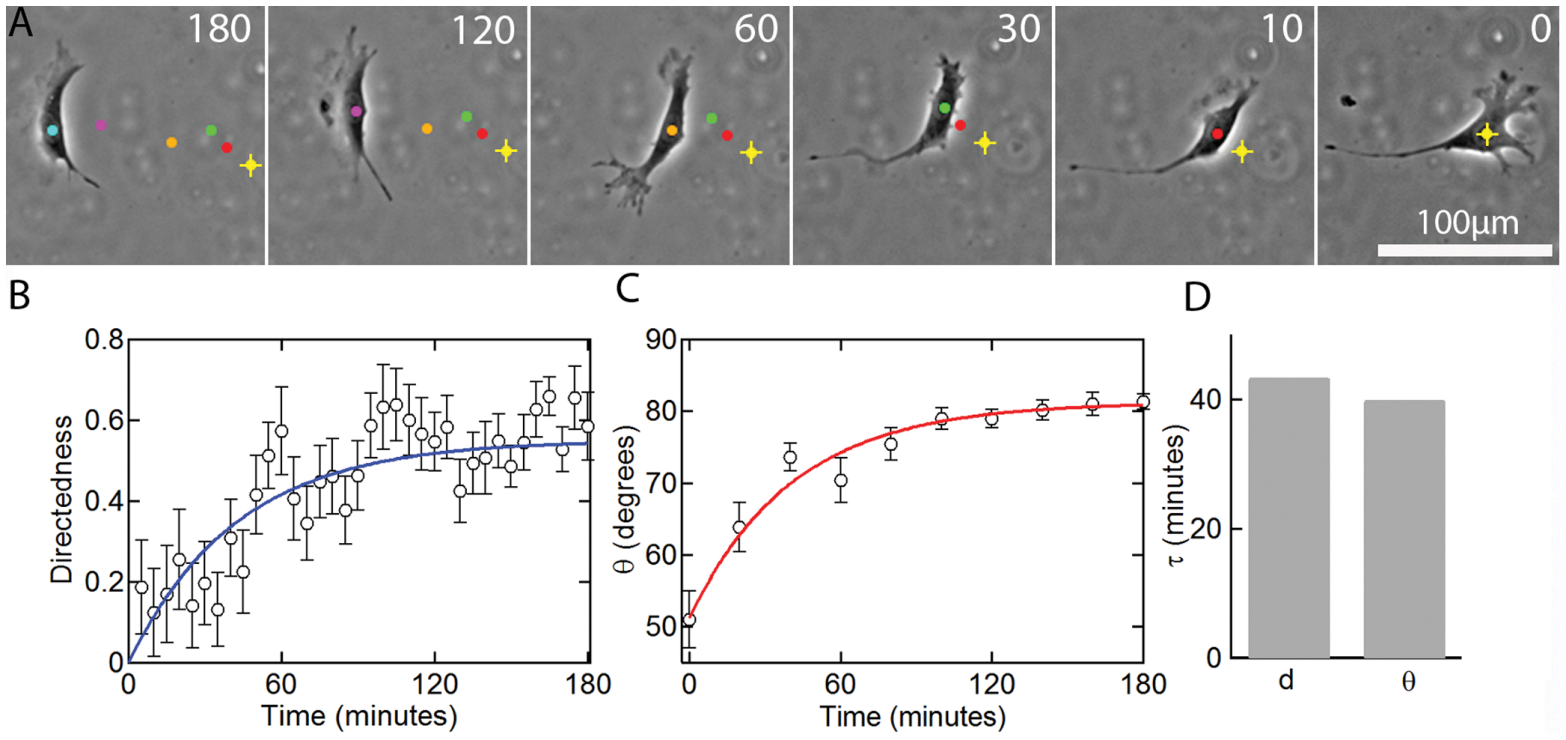
Response to an electric field in 2D. (A) Time-lapse images showing the reorientation of a 3T3 cell in a 5.5 V cm^−1^ electric field. The time (min) after applying the electric field is indicated in the images. (B) The average directedness (d) of 3T3 cells versus time after applying a 5.5 V cm^−1^ field. (C) The average cell orientation (θ) versus time after applying a 5.5 V cm^−1^ field (N = 36). The solid lines represent fits to an equation of the form (x−x_min_)/(x_max_−x_min_) = 1−exp(−t/τ) where x = d or θ and τ is the time constant. For the directedness, d_min_ = 0 and d_max_ is the average directedness from 120–180 min. (D) Time constants for the transient change in directedness and cell orientation after applying a 5.5 V cm^−1^ field.

In addition to the steady state behavior, we also studied the transient response to an electric field. The key parameters in the transient response are the directedness and the overall cell orientation. In the absence of confinement, on application of a 5.5 V cm^−1^ electric field, cell re-orientation occurs over about 2 hours (Fig. 5A). The time-lapse images show that cells first send out protrusions in the desired locations to align perpendicular to the field vector before developing stable cathode-facing lamellipodia. The time dependence of the average directedness of the cells follows an exponential increase (Fig. 5B) with a time constant τ of 43.5 minutes (Fig. 5D). Similarly, the average cell orientation angle (θ_avg_) increases exponentially (Fig. 5C) with a time constant τ of 40 minutes (Fig. 5D). The progressively smaller error bars indicate that cell orientation gradually converges to a steady state perpendicular to the field vector. Cell orientation also increased exponentially in the presence of a 2.2 V cm^−1^ field (Fig. S5); however, cells aligned substantially slower as suggested by a greater time constant (τ = 95 minutes).

### Conclusions

Through detailed analysis of cell paths we show that the influence of electric field on motility is much more complex than simply imposing a directional bias towards the cathode or anode. External electric fields with magnitude similar to endogenous electric field modulate several processes within 3T3 cells, involving overall cell reorientation, and motion both parallel and perpendicular to the field. Detailed analysis of cell paths reveal forces both parallel and perpendicular to the electric field that drives migration in a field dependent manner. Confinement in 2D channel prevents cell orientation perpendicular to the field and results in increased cell velocity. These results provide new insight into the biophysical response of cells to electric fields that can guide further research into the signaling pathways that regulate galvanotaxis.

## Supporting Information

Figure S1 **Analysis of cell trajectories base on mean square displacement (MSD).** (A) Overlay of the MSD for each cell in a single experiment. (B) Effect of unweighted (UW) and weighted (W) persistent random walk (PRW) on the fit. The weighted PRW method put more emphasis on the initial points (smaller time intervals). (C) Instantaneous velocities (V-inst) are extrapolated from the average velocity calculated from the trajectories (V-traj) to Δt = 0. (D) Comparison of the average velocity (V-traj) and instantaneous velocity (V-inst) to root-mean square speed calculated from different methods under no confinement. U/A: unweighted average then fit, U/F: unweighted fit then average, W/A: weighted average then fit, W/F: weighted fit then average (W/F). (E) Comparison of V-traj and V-inst to root-mean square speeds in 20 µm channel. (F) Comparison of persistence time (P) base on arbitrarily defining the persistence as time of migration where δ >70° and four variations of PRW model mentioned above.
(TIF)

Figure S2 **α_v_ versus average velocity in 2D (open symbols) and within 20 µm channels (solid symbols).** A linear relationship that goes through the origin exists in the case of 2D but not under confinement. (square: control, circle: 2.2 V cm^−1^, triangle: 5.5 V cm^−1^)
(TIF)

Figure S3 **Scatter plots of the x- and y-components of each segment in no field (A), a 2.2** **V** **cm^−1^ field (B), and a 5.5** **V cm ^−1^ field (C).** In the absence of a field, the scatter plot is symmetrical about the origin (A). In a 2.2 V cm^−1^ field, Δy has a greater effect on cell motion and therefore, the x- and y-components of each segment are scattered along the y-axis with a higher frequency between 60° to 90° and −60° to −90° (B). In a 5.5 V cm^−1^ field, horizontal bias toward the cathode becomes dominant and the majority of the x- and y-components are within ±45° (C).
(TIF)

Figure S4 **Distributions of average cell velocity, segment orientation (θ), and segment turn angle (δ) in 20 µm channels with no field (A, B, C), in a 2.2** **V cm^−1^ field (D, E, F), and in a 5.5** **V cm^−1^ field (G, H, I).** The distribution of velocity is exponential in the absence of an electric field (R^2^ = 0.88) (A), in a 2.2 V cm^−1^ (R^2^ = 0.89) (D), and in a 5.5 V cm^−1^ field (R^2^ = 0.92) (G). (B) In the absence of a field, the distribution of segment angles is bipolar and symmetrical with peaks at θ = 0° and 180°. (C) In the absence of a field, the segment turn angle remains bipolar but with a large exponential distribution around 0° and a small distribution around 180°. (D) Distribution of velocity remains exponential in a 2.2 V cm^−1^ field. (E) In the presence of a field, the approximately symmetrical distribution of δ becomes strongly biased towards small angles. (F) There is relatively little change in the distribution of segment turn angles in the presence of a field. (G) Further increasing the electric filed to 5.5 V cm^−1^, significantly increases the strength of the exponential, α_v_ increases from 0.5 to 1.2 µm min^−1^. (H and I) No further changes in the distribution of segment orientation and segment turn angle were seen in the presence of a 5.5 V cm^−1^ field comparing to a no field. (J) Comparison of α_v_ between no confinement (black) and confinement (blue). (K) Confinement greatly decreases α_δ._ Similar to the results in 2D, electric field has no obvious effect on α_δ_ under confinement.
(TIF)

Figure S5 **Transient response of cell orientation in a 2.2** **V cm^−1^ field.** The cell orientation (θ) increased exponentially in the presence of a 2.2 V cm^−1^ field; however, the time constant τ = 95 minutes, much longer than in a 5.5 V cm ^−1^ field where τ = 40 minutes.
(TIF)

Supporting Information S1 (DOCX)

Video S1 **3T3 cells under no confinement and no field.**
(MP4)

Video S2 **3T3 cells under no confinement but in the presence of a 5.5 V cm^−1^ field.**
(MP4)

Video S3 **3T3 cells confined in 20 µm channels in the absence of a field.**
(MP4)

Video S4 **3T3 cells confined in 20 µm channels in the presence of a 5.5 V cm^−1^ field.**
(MP4)

## References

[1] Mycielska ME, Djamgoz MBA (2004) Cellular mechanisms of direct-current electric field effects: galvanotaxis and metastatic disease. Journal of Cell Science 117: 1631–1639. 15075225 10.1242/jcs.01125

[2] Nuccitelli R (2003) A role for endogenous electric fields in wound healing. Curr Top Dev Biol 58: 1–26. 14711011 10.1016/s0070-2153(03)58001-2

[3] McCaig CD, Rajnicek AM, Song B, Zhao M (2005) Controlling cell behavior electrically: Current views and future potential. Physiological Reviews 85: 943–978. 15987799 10.1152/physrev.00020.2004

[4] Weaver JC, Chizmadzhev YA (1996) Theory of electroporation: A review. Bioelectrochemistry and Bioenergetics 41: 135–160.

[5] Zhao M, Song B, Pu J, Wada T, Reid B, et al (2006) Electrical signals control wound healing through phosphatidylinositol-3-OH kinase-gamma and PTEN. Nature 442: 457–460. 16871217 10.1038/nature04925

[6] Song B, Zhao M, Forrester JV, McCaig CD (2002) Electrical cues regulate the orientation and frequency of cell division and the rate of wound healing in vivo. Proceedings of the National Academy of Sciences of the United States of America 99: 13577–13582. 12368473 10.1073/pnas.202235299 PMC129716

[7] Hotary KB, Robinson KR (1992) Evidence of a role for endogenous electrical fields in chick embryo development. Development 114: 985–996. 1618158 10.1242/dev.114.4.985

[8] Hotary KB, Robinson KR (1994) Endogenous electrical currents and voltage gradients in Xenopus embryos and the consequences of their disruption. Dev Biol 166: 789–800. 7813796 10.1006/dbio.1994.1357

[9] Friedl P, Alexander S (2011) Cancer invasion and the microenvironment: plasticity and reciprocity. Cell 147: 992–1009. 22118458 10.1016/j.cell.2011.11.016

[10] Song B, Gu Y, Pu J, Reid B, Zhao ZQ, et al (2007) Application of direct current electric fields to cells and tissues in vitro and modulation of wound electric field in vivo. Nature Protocols 2: 1479–1489. 17545984 10.1038/nprot.2007.205

[11] Huang CW, Cheng JY, Yen MH, Young TH (2009) Electrotaxis of lung cancer cells in a multiple-electric-field chip. Biosensors & Bioelectronics 24: 3510–3516. 19497728 10.1016/j.bios.2009.05.001

[12] Wang CC, Kao YC, Chi PY, Huang CW, Lin JY, et al (2011) Asymmetric cancer-cell filopodium growth induced by electric-fields in a microfluidic culture chip. Lab Chip 11: 695–699. 21152515 10.1039/c0lc00155d

[13] Li J, Lin F (2011) Microfluidic devices for studying chemotaxis and electrotaxis. Trends Cell Biol 21: 489–497. 21665472 10.1016/j.tcb.2011.05.002

[14] Li J, Nandagopal S, Wu D, Romanuik SF, Paul K, et al (2011) Activated T lymphocytes migrate toward the cathode of DC electric fields in microfluidic devices. Lab Chip 11: 1298–1304. 21327249 10.1039/c0lc00371a

[15] Li XF, Kolega J (2002) Effects of direct current electric fields on cell migration and actin filament distribution in bovine vascular endothelial cells. Journal of Vascular Research 39: 391–404. 12297702 10.1159/000064517

[16] Wirtz D, Konstantopoulos K, Searson PC (2011) The physics of cancer: the role of physical interactions and mechanical forces in metastasis. Nature Reviews Cancer 11: 512–522. 21701513 10.1038/nrc3080 PMC3262453

[17] Fraley SI, Feng Y, Krishnamurthy R, Kim DH, Celedon A, et al (2010) A distinctive role for focal adhesion proteins in three-dimensional cell motility. nature cell biology 12: 598–604. 20473295 10.1038/ncb2062 PMC3116660

[18] Shoemaker DP, Garland CW, Nibler JW, Feigerle CS (1967) Experiments in physical chemistry: McGraw-Hill New York.

[19] Walmod PS, Hartmann-Petersen R, Berezin A, Prag S, Kiselyov VV, et al (2001) Evaluation of individual-cell motility. Methods Mol Biol 161: 59–83. 11190517 10.1385/1-59259-051-9:059

[20] Cooper M, Keller R (1984) Perpendicular orientation and directional migration of amphibian neural crest cells in dc electrical fields. Proceedings of the National Academy of Sciences 81: 160. 10.1073/pnas.81.1.160 PMC344630 6582473

[21] Hammerick KE, Longaker MT, Prinz FB (2010) In vitro effects of direct current electric fields on adipose-derived stromal cells. Biochem Biophys Res Commun 397: 12–17. 20452327 10.1016/j.bbrc.2010.05.003 PMC5718355

[22] Finkelstein E, Chang W, Chao PH, Gruber D, Minden A, et al (2004) Roles of microtubules, cell polarity and adhesion in electric-field-mediated motility of 3T3 fibroblasts. J Cell Sci 117: 1533–1545. 15020680 10.1242/jcs.00986

[23] Kalluri R, Zeisberg M (2006) Fibroblasts in cancer. Nature Reviews Cancer 6: 392–401. 16572188 10.1038/nrc1877

[24] Brown MJ, Loew LM (1994) Electric field-directed fibroblast locomotion involves cell surface molecular reorganization and is calcium independent. J Cell Biol 127: 117–128. 7929557 10.1083/jcb.127.1.117 PMC2120190

[25] Dunn GA, Brown AF (1987) A Unified Approach to Analyzing Cell Motility. Journal of Cell Science: 81–102. 10.1242/jcs.1987.supplement_8.5 3503898

[26] Stokes CL, Lauffenburger DA, Williams SK (1991) Migration of individual microvessel endothelial cells: stochastic model and parameter measurement. J Cell Sci 99 (Pt 2): 419–430. 10.1242/jcs.99.2.419 1885678

[27] Czirok A, Schlett K, Madarasz E, Vicsek T (1998) Exponential distribution of locomotion activity in cell cultures. Physical Review Letters 81: 3038–3041.

[28] Selmeczi D, Mosler S, Hagedorn PH, Larsen NB, Flyvbjerg H (2005) Cell motility as persistent random motion: Theories from experiments. Biophysical Journal 89: 912–931. 15951372 10.1529/biophysj.105.061150 PMC1366641

[29] Li L, Norrelykke SF, Cox EC (2008) Persistent Cell Motion in the Absence of External Signals: A Search Strategy for Eukaryotic Cells. PLoS One 3: e2093. 18461173 10.1371/journal.pone.0002093 PMC2358978

[30] Doyle AD, Wang FW, Matsumoto K, Yamada KM (2009) One-dimensional topography underlies three-dimensional fibrillar cell migration. J Cell Biol 184: 481–490. 19221195 10.1083/jcb.200810041 PMC2654121

[31] Balzer EM, Tong Z, Paul CD, Hung W-C, Stroka KM, et al (2012) Physical confinement alters tumor cell adhesion and migration phenotypes. The FASEB Journal 26: 4045–4056. 22707566 10.1096/fj.12-211441 PMC3448771

[32] Tong ZQ, Balzer EM, Dallas MR, Hung WC, Stebe KJ, et al (2012) Chemotaxis of cell populations through confined spaces at single-cell resolution. PLoS One 7: e29211. 22279529 10.1371/journal.pone.0029211 PMC3261140

[33] Hawkins RJ, Piel M, Faure-Andre G, Lennon-Dumenil A, Joanny J, et al (2009) Pushing off the walls: a mechanism of cell motility in confinement. Physical review letters 102: 58103. 10.1103/PhysRevLett.102.058103 19257561

