# Supplementary figures and images for: The Influence of Electric Field and Confinement on Cell Motility
Source: PLoS One. 2013 Mar 26;8(3):e59447. doi: 10.1371/journal.pone.0059447 (PMC3608730; doi:10.1371/journal.pone.0059447)

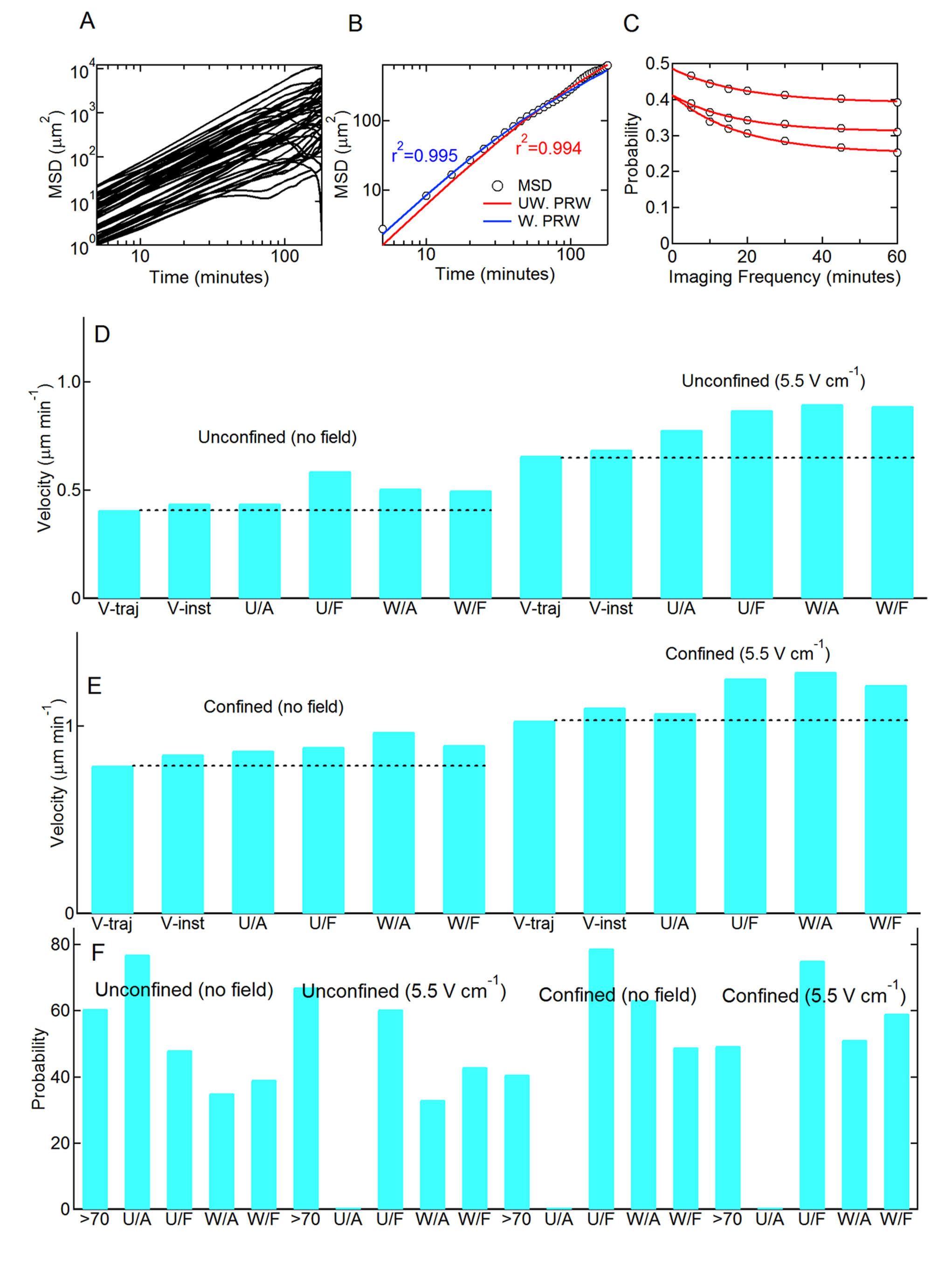

Supplement: Figure S1 — Analysis of cell trajectories base on mean square displacement (MSD). (A) Overlay of the MSD for each cell in a single experiment. (B) Effect of unweighted (UW) and weighted (W) persistent random walk (PRW) on the fit. The weighted PRW method put more emphasis on the initial points (smaller time intervals). (C) Instantaneous velocities (V-inst) are extrapolated from the average velocity calculated from the trajectories (V-traj) to Δt = 0. (D) Comparison of the average velocity (V-traj) and instantaneous velocity (V-inst) to root-mean square speed calculated from different methods under no confinement. U/A: unweighted average then fit, U/F: unweighted fit then average, W/A: weighted average then fit, W/F: weighted fit then average (W/F). (E) Comparison of V-traj and V-inst to root-mean square speeds in 20 µm channel. (F) Comparison of persistence time (P) base on arbitrarily defining the persistence as time of migration where δ >70° and four variations of PRW model mentioned above. (TIF) [file pone.0059447.s001.tif]

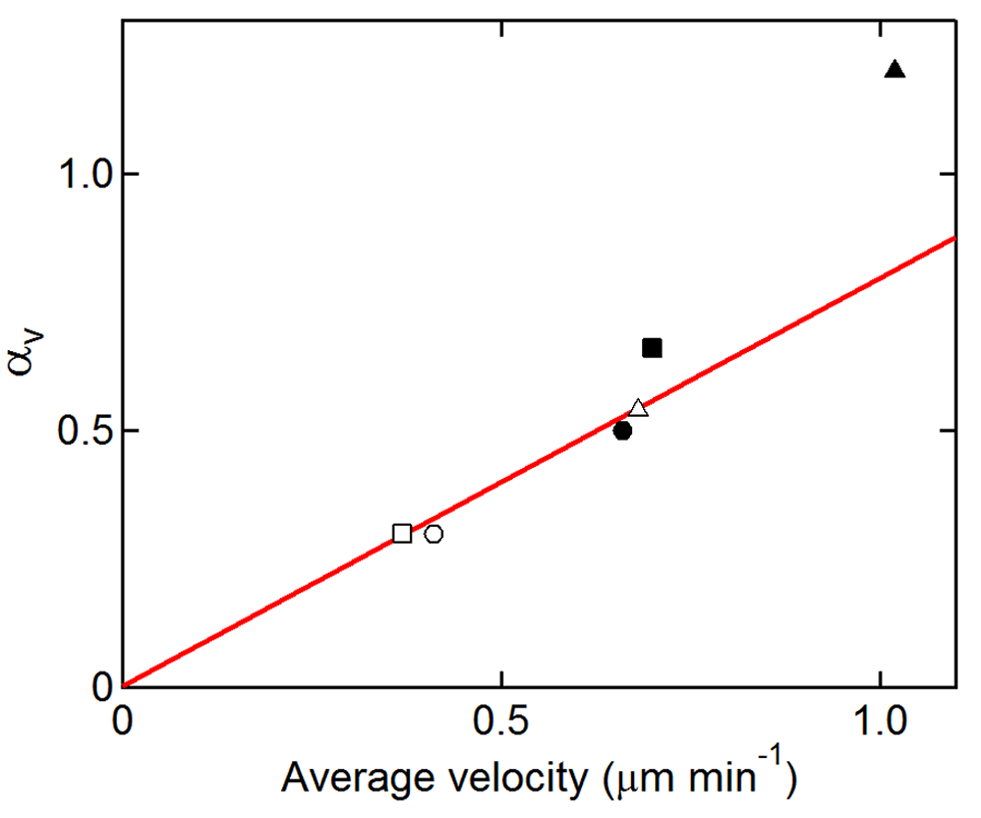

Supplement: Figure S2 — αv versus average velocity in 2D (open symbols) and within 20 µm channels (solid symbols). A linear relationship that goes through the origin exists in the case of 2D but not under confinement. (square: control, circle: 2.2 V cm−1, triangle: 5.5 V cm−1) (TIF) [file pone.0059447.s002.tif]

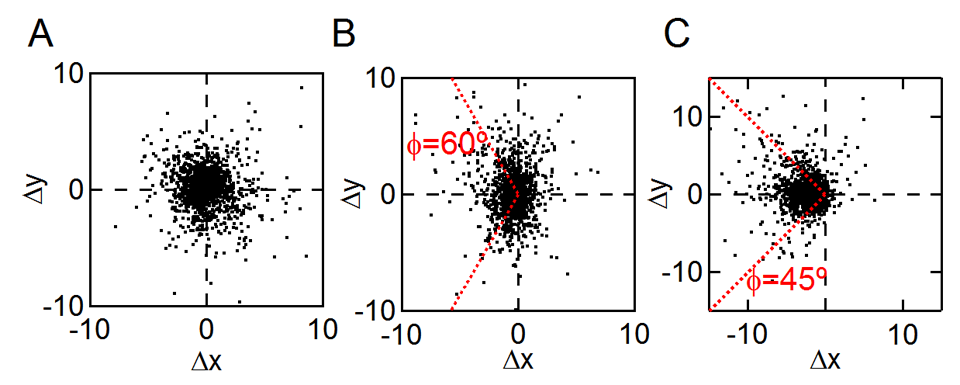

Supplement: Figure S3 — Scatter plots of the x- and y-components of each segment in no field (A), a 2.2 V cm−1 field (B), and a 5.5 V cm −1 field (C). In the absence of a field, the scatter plot is symmetrical about the origin (A). In a 2.2 V cm−1 field, Δy has a greater effect on cell motion and therefore, the x- and y-components of each segment are scattered along the y-axis with a higher frequency between 60° to 90° and −60° to −90° (B). In a 5.5 V cm−1 field, horizontal bias toward the cathode becomes dominant and the majority of the x- and y-components are within ±45° (C). (TIF) [file pone.0059447.s003.tif]

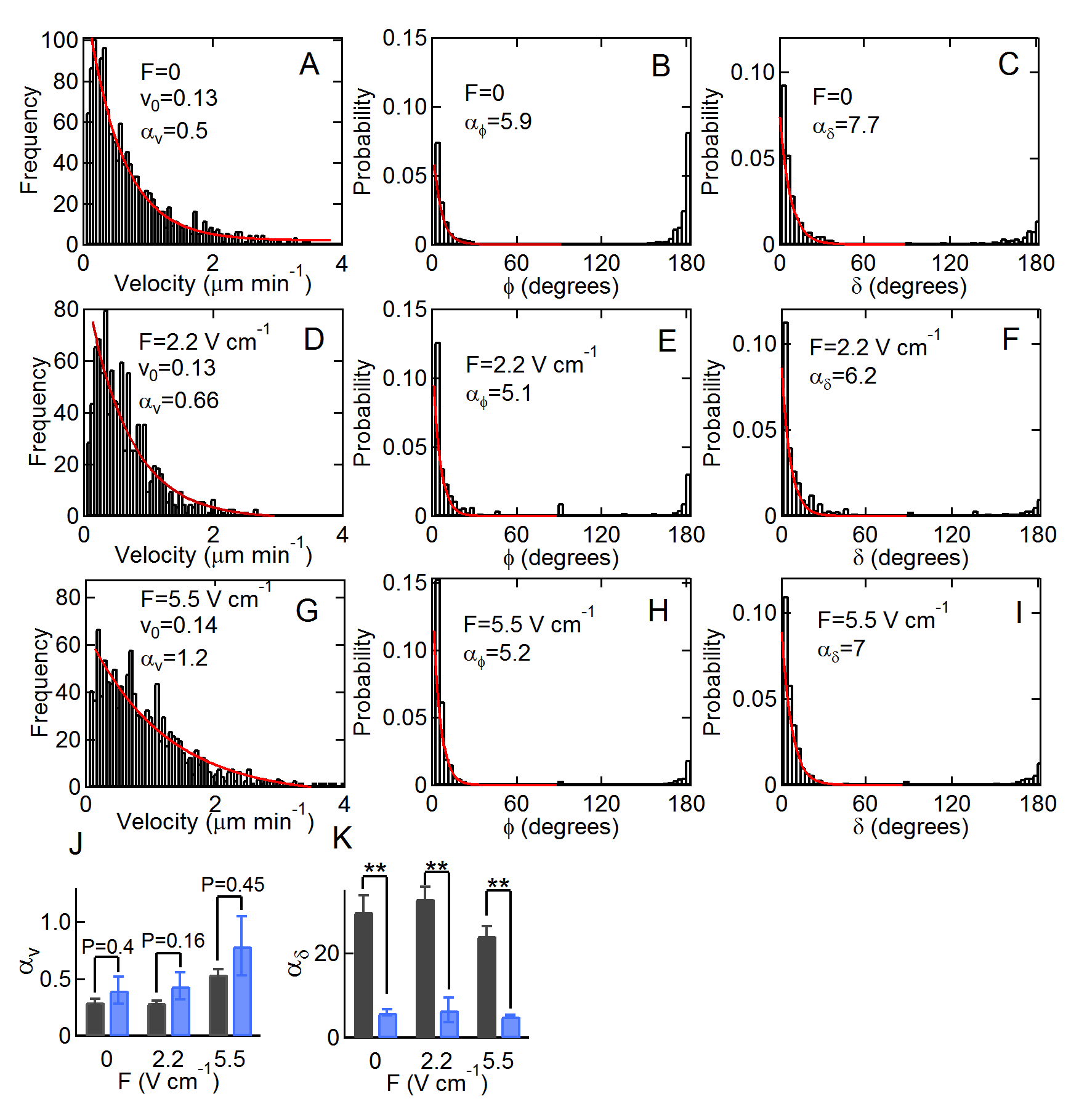

Supplement: Figure S4 — Distributions of average cell velocity, segment orientation (θ), and segment turn angle (δ) in 20 µm channels with no field (A, B, C), in a 2.2 V cm−1 field (D, E, F), and in a 5.5 V cm−1 field (G, H, I). The distribution of velocity is exponential in the absence of an electric field (R2 = 0.88) (A), in a 2.2 V cm−1 (R2 = 0.89) (D), and in a 5.5 V cm−1 field (R2 = 0.92) (G). (B) In the absence of a field, the distribution of segment angles is bipolar and symmetrical with peaks at θ = 0° and 180°. (C) In the absence of a field, the segment turn angle remains bipolar but with a large exponential distribution around 0° and a small distribution around 180°. (D) Distribution of velocity remains exponential in a 2.2 V cm−1 field. (E) In the presence of a field, the approximately symmetrical distribution of δ becomes strongly biased towards small angles. (F) There is relatively little change in the distribution of segment turn angles in the presence of a field. (G) Further increasing the electric filed to 5.5 V cm−1, significantly increases the strength of the exponential, αv increases from 0.5 to 1.2 µm min−1. (H and I) No further changes in the distribution of segment orientation and segment turn angle were seen in the presence of a 5.5 V cm−1 field comparing to a no field. (J) Comparison of αv between no confinement (black) and confinement (blue). (K) Confinement greatly decreases αδ. Similar to the results in 2D, electric field has no obvious effect on αδ under confinement. (TIF) [file pone.0059447.s004.tif]

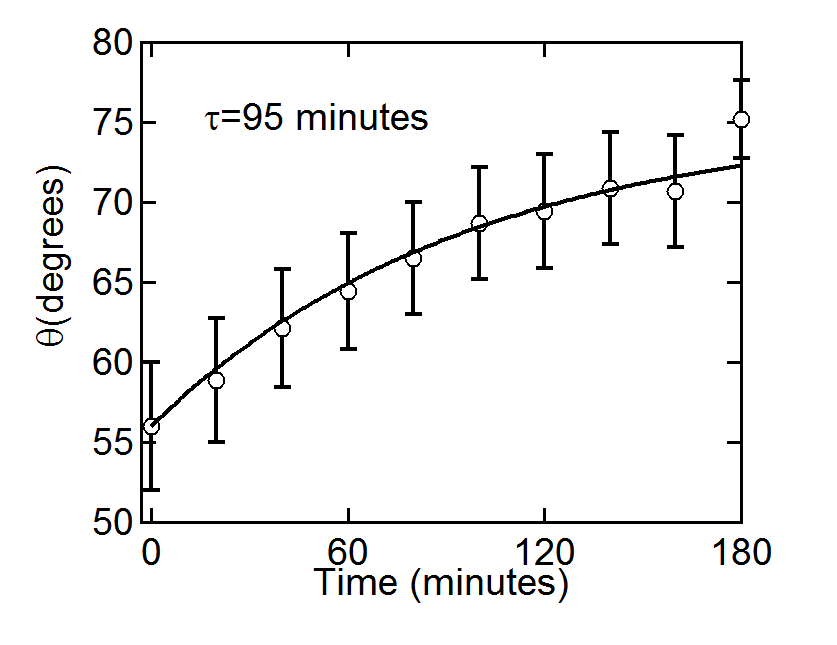

Supplement: Figure S5 — Transient response of cell orientation in a 2.2 V cm−1 field. The cell orientation (θ) increased exponentially in the presence of a 2.2 V cm−1 field; however, the time constant τ = 95 minutes, much longer than in a 5.5 V cm −1 field where τ = 40 minutes. (TIF) [file pone.0059447.s005.tif]
