# Supplementary material for: The Influence of Electric Field and Confinement on Cell Motility
Source: PLoS One. 2013 Mar 26;8(3):e59447. doi: 10.1371/journal.pone.0059447 (PMC3608730; doi:10.1371/journal.pone.0059447)
Supplement: Supporting Information S1 — (DOCX) [file pone.0059447.s006.docx]

# The Influence of Electric Field and Confinement on Cell Motility

# Yu-Ja Huang, Justin Samorajski, Rachel Kreimer, and Peter C. Searson

# Johns Hopkins University, Baltimore, Maryland, USA

# Supporting information

Fabrication of Microfluidic Platform

Platform Integration

Analysis of Mean Square Displacement

Figure S1: Analysis of cell trajectories based on the mean square displacement (MSD).

Figure S2: α_v_ versus average velocity in 2D and in 20 μm channels

Figure S3: Scatter plots of the x- and y-components of each segment in the absence of a field, under a 2.2 V cm^-1^ field, and under a 5.5 V cm ^-1^ field.

Figure S4: Distributions of average cell velocity, segment orientation (θ), and segment turn angle (δ) in 20 µm channels with no field, in a 2.2 V cm^-1^ field, and in a 5.5 V cm^-1^ field.

Figure S5: Transient response of cell orientation in a 2.2 V cm^-1^ field.

Video S1: 3T3 cells under no confinement and no field.

Video S2: 3T3 cells under no confinement in the presence of a 5.5 V cm^-1^ field.

Video S3: 3T3 cells confined in 20 μm channels in the absence of a field.

Video S4: 3T3 cells confined in 20 μm channels in the presence of a 5.5 V cm^-1^ field.

# Fabrication of Microfluidic Platform

The galvanotaxis platform includes a central microfluidic channel or channels located between two media reservoirs and integrated electrode ports. A separate cell injection port is located at one end of the microfluidic channel. To fabricate the mold for the device, an 80 µm thick photoresist film (SU-8 2075, Microchem, Newton, MA) was first spin coated on a silicon wafer. The photoresist was then exposed in a mask aligner (OAI, San Jose, CA) using a custom-designed mask. Next, a second 80 µm thick photoresist layer was spin coated onto the first layer and after alignment was exposed so that the mold was 160 µm thick everywhere but 80 µm thick in the main channel. Next, the unexposed photoresist was removed using SU-8 developer (Microchem, Newton, MA).

Devices were fabricated by pouring polydimethysiloxane (PDMS) (Dow Corning, Midland, MI) on the mold and curing at 65 ˚C. Subsequently, nylon tubes (part 91145A161, McMaster, Robbinsville, NJ) that served as reservoirs, were carefully located at the desired locations before the second layer of PDMS was cast and cured over the mold.

The inlets and outlets, located in the center of each reservoir, were formed using a 2.5 mm punch (Harris Uni-core, Ted Pella, Redding, CA). The inlet of the cell injection port was created by punching through the PDMS with a 30-gauge blunt head needle (Fisnar, Wayne, NJ). Each PDMS device was bonded to a No. 1 coverslip (Fisher Scientific, Pittsburgh, PA) by exposing to an oxygen plasma for 50 seconds (Plasma Etch, Carson, Nevada). All experiments reported here were performed in a 1000 µm channel (no confinement, 2D) or a 20 µm channel.

# Platform Integration

Immediately after assembly, the microfluidic platform was placed in a sterile tissue culture hood. The main channel was incubated with 200 µL of 50 μg mL^-1^ FITC conjugated fibronectin (BD) solution for one hour. The device was then washed with phosphate buffer (PBS, Gibco) and the reservoirs and channel filled with cell media.

The Ag/AgCl electrodes were integrated directly into the device by embedding in agarose gel. This approach allows us to avoid conventional salt bridges and external solution reservoirs containing the electrodes. 2 wt.% of cell-culture grade agarose powder (Invitrogen) was dissolved in Steinberg’s solution by gently heating the solution at 130 ˚C. Prior to adding the agarose solution into the electrode reservoirs, the media reservoirs were capped with rubber septa to create a positive pressure that prevented agarose from flowing into the channels during gelation. 500 µL of the agarose solution was added to each electrode reservoir. A Ag/AgCl electrode was then immediately inserted into the agarose solution and the electrode port was sealed with a centrifugal filter (Millipore, Billerica, MA) to ensure a good contact between the electrode and agarose and to prevent evaporation. The devices were then left in a tissue culture hood for 30 minutes to ensure the agarose had completely gelled. A constant voltage was applied by connecting two Ag/AgCl electrodes embedded in agarose to a potentiostat (VersaStat 3, Princeton Applied Research). The actual voltage drop across both ends of the channel was measured using a four-point probe method with two platinum wires. The electric field was further verified based on the resistivity of the media inside the channel and the current recorded by the potentiostat.

# Analysis of Mean Square Displacement

The mean square displacements (MSD) for individual cells were determined from the experimentally acquired cell paths using over-lapping intervals [[1](#_ENREF_1)]. The persistent random walk (PRW) model is commonly used to analyze cell motility in 2D [[2-4](#_ENREF_2)]. This model is described by: $\left\langle d^{2}\right\rangle=2S^{2}P\left(t-P\left(1-\exp\left(-\frac{t}{P}\right)\right)\right)$ (1)

where <d^2^> is the mean square displacement (MSD), S is the root-mean square speed, and P is the directional persistence time [[2](#_ENREF_2)]. Several modifications to further improve the applicability and consistency of the model have been proposed, however, there is no standard way of analyzing MSDs [[5](#_ENREF_5)]. The MSD of all cells can first be averaged and then fit to the PRW model or first be fit to the PRW model and then averaged later. Here we present the results based on four common variations for applying the PRW model: unweighted average then fit (U/A), unweighted fit then average (U/F), weighted average then fit (W/A), and weighted fit then average (W/F). The weighting was used to account for the increasingly lower numbers of points at longer time intervals [[1](#_ENREF_1),[6](#_ENREF_6)]. In other words, the weighted model puts more emphasis on the initial points (smaller time interval) as shown in Figure S1B.

The results of the analysis of the cell paths using the PRW model are summarized in Figure S1. For cell motility in 2D in the absence of a field, the values of S obtained from the MSDs were slightly larger than the values obtained from analysis of the individual segments, but were all within 20% with the exception of the U/F method. The best agreement between the average cell velocity obtained from the individual segments and the MSDs was obtained by calculating the unweighted average of all the MSDs and then fitting to equation (1). In the absence of confinement and electric field, the average cell velocity obtained from analysis of the individual segments was 0.37 µm min^-1^ (Fig. 3F) and from the MSDs was 0.44 µm min^-1^ (Fig. S1D, U/A). The cell velocity obtained from analysis of the individual segments was obtained for ∆t = 5 minutes and was weakly dependent on sampling time, as has been reported previously [[7](#_ENREF_7)]. Extrapolation of the average cell velocity to ∆t = 0 (Fig. S1C) gives the instantaneous velocity of 0.44 µm min^-1^, in excellent agreement with the value obtained from analysis of the MSDs.

In the presence of an electric field, cell velocities obtained from MSDs were on average 30% higher than the value obtained from the individual segments with the U/A method giving the best agreement (18%). The difference further decreased when comparing to the instantaneous velocity.

Interestingly, in 20 µm channels, the cell velocities obtained from MSDs had better agreement with values obtained from analysis of individual segments. The average differences were 14% (no field) and 17% (5.5 V cm^-1^ field), respectively. When compared to the instantaneous velocity, the difference further decreased to 6% and 10%, respectively. Note that, the U/A method still yields the best result among the four methods tested.

In addition to the average cell speed S, the directional persistence time P can also be extracted from analysis of the MSDs. Values for P obtained from fits to equation (1) (Fig. S1) varied between 30 - 80 minutes. In general, smaller values for P were obtained using weighted individual (W/F) or averaged (W/A). Comparison of the results shows no obvious influence of electric field or confinement. Similar results were obtained by arbitrarily defining the persistence as the time of migration where δ < 70˚ (Fig. S1).

# References

1. Qian H, Sheetz MP, Elson EL (1991) Single particle tracking. Analysis of diffusion and flow in two-dimensional systems. Biophysical journal 60: 910-921.

2. Dunn GA, Brown AF (1987) A Unified Approach to Analyzing Cell Motility. Journal of Cell Science: 81-102.

3. Walmod PS, Hartmann-Petersen R, Berezin A, Prag S, Kiselyov VV, et al. (2001) Evaluation of individual-cell motility. Methods Mol Biol 161: 59-83.

4. Stokes CL, Lauffenburger DA, Williams SK (1991) Migration of individual microvessel endothelial cells: stochastic model and parameter measurement. J Cell Sci 99 ( Pt 2): 419-430.

5. Selmeczi D, Mosler S, Hagedorn PH, Larsen NB, Flyvbjerg H (2005) Cell motility as persistent random motion: Theories from experiments. Biophysical Journal 89: 912-931.

6. Li L, Norrelykke SF, Cox EC (2008) Persistent Cell Motion in the Absence of External Signals: A Search Strategy for Eukaryotic Cells. PLoS One 3: e2093.

7. Potdar AA, Lu J, Jeon J, Weaver AM, Cummings PT (2009) Bimodal analysis of mammary epithelial cell migration in two dimensions. Annals of Biomedical Engineering 37: 230-245.
